# Association between serum vitamin A levels and premature ovarian insufficiency: a case–control, cross-sectional survey study

**DOI:** 10.1186/s12902-022-01003-9

**Published:** 2022-04-04

**Authors:** Peiqiong Chen, Yang Song, Wenxian Xu, Yizhou Huang, Yingxian Jia, Chunming Li, Yibing Lan, Ketan Chu, Linjuan Ma, Jianhong Zhou

**Affiliations:** Department of Gynecology, Women’s Hospital, Zhejiang University School of Medicine, First Xueshi Rd, Hangzhou, 310006 People’s Republic of China

**Keywords:** Vitamin A, Retinoid signaling, Premature ovarian insufficiency, Anti-Müllerian hormone

## Abstract

**Background:**

Although vitamin A is known to play an important role in ovarian function, its association with ovarian insufficiency has not been reported yet. Therefore, the aim of the study was to explore the association between serum vitamin A levels and premature ovarian insufficiency (POI).

**Methods:**

This cross-sectional survey included women with POI (*n* = 47) and normo-ovulatory controls (*n* = 67) who were enrolled between December 2016 and May 2018 in Zhejiang, China. The serum levels of follicle-stimulating hormone (FSH), luteinizing hormone (LH), estradiol (E_2_), anti-Müllerian hormone (AMH), vitamin A, and total cholesterol (TC) were measured for each participant. The association of TC-adjusted vitamin A levels with the risk of POI was assessed using binary logistic regression analysis.

**Results:**

Serum vitamin A levels appeared to be slightly higher in the POI group than in the control group, but there was no evidence of a statistically significant difference (728.00 ± 176.00 µg/L vs. 503.93 ± 145.64 µg/L, *p* = 0.13). After adjustment for serum lipid levels, the serum vitamin A/TC ratio was significantly lower in the POI group than in the control group (143.14 ± 35.86 vs. 157.56 ± 35.21 µg/mmol, *p* = 0.04). Further, the serum vitamin A/TC ratio was significantly and inversely associated with POI risk (unadjusted odds ratio [OR] = 0.988, 95% confidence interval [CI]: 0.977–0.999, *p* = 0.04). The association remained after adjusting for confounding factors (age, BMI, annual household income, and education) (OR = 0.986, 95% CI: 0.972–0.999, *p* = 0.04).

**Conclusions:**

Serum vitamin A/TC ratio was inversely associated with POI risk. Therefore, the serum vitamin A/TC ratio may serve as a predictive factor for POI, and vitamin A supplementation may play help prevent or treat POI.

## Background

Premature ovarian insufficiency (POI) currently affects 1% of the female population globally [1]. POI is manifested as ovarian dysfunction and infertility, and is associated with an increased risk of mortality, cancer, cardiovascular disease, and osteoporosis [2, 3]. There has been much concern about ovarian insufficiency over the past few decades [4], and studies have shown that POI may be correlated with chromosomal abnormalities, environmental pollutants, social stress, postponement of childbearing, autoimmunity, and prolonged survival of patients after gonadotoxic treatments such as paclitaxel treatment [1, 5, 6]. However, in 90% of POI cases, the causative factor is unclear [4]. Therefore, there is a need for more studies on the etiology of POI.

Vitamin A (retinol), a fat-soluble vitamin, is essential for reproduction, and retinol deficiency or excess may result in embryonic loss and/or congenital defects [7–9]. Studies have shown that the vitamin A signaling pathway has potential effects on follicular development, oocyte maturation, ovarian steroidogenesis, and corpus luteum formation [10–12]. Moreover, studies have shown that the vitamin A level in follicular fluid is associated with the quality of human oocytes, fertilization potential, and embryo development [13]. However, vitamin A deficiency (VAD) is common in women of reproductive age, and even more common in pregnant women. In particular, in low-income countries, 10%–20% of pregnant women develop VAD [14]. Although many studies have confirmed the role of vitamin A in ovarian physiological function, the serum vitamin A levels in women with POI have seldom been considered before. Therefore, the aim of the present study was to explore the association between serum vitamin A levels and POI risk in women of reproductive age.

This case–control, cross-sectional study was conducted on young women diagnosed with POI and normo-ovulatory controls matched by age and body mass index (BMI). To the best of our knowledge, this is the first study to report an association between vitamin A levels and POI in women of reproductive age.

## Materials and methods

### Study population

This study was performed at the Department of Gynecology, Women’s Hospital, School of Medicine, Zhejiang University, Hangzhou, Zhejiang, China. A total of 47 patients diagnosed with POI between December 2014 and May 2018 were identified from laboratory records and medical history (age, mean [range]: 33.30 ± 6.45 [18–42] years). Eight women were above 40 years old at the time of enrollment but were diagnosed with POI before the age of 40 years. The control group, selected based on the results of a health examination, included 67 women of reproductive age (age, mean [range]: 32.63 ± 5.87 [21–43] years) with normal menstrual cycles. The study protocol was in accordance with the Declaration of Helsinki and was approved by the ethics committee of Women’s Hospital, School of Medicine, Zhejiang University. Informed consent was obtained from all the participants.

### Definitions

POI was diagnosed based on the criteria of the European Society of Human Reproduction and Embryology [15]. The eligibility criteria for this study included oligomenorrhea or amenorrhea for at least 4 months, elevated follicle-stimulating hormone (FSH) levels (> 25 IU/L) on two measurements taken more than 4 weeks apart, and age below 40 years at the time of the first diagnosis. The control group was recruited based on routine physical check-ups and matched with the POI group based on BMI and age (± 3 years).

The exclusion criteria were autoimmune diseases, chronic diseases (liver diseases, renal diseases, hypertension, cardiovascular or cerebrovascular diseases, and congestive heart failure), and a history of hysterectomy, oophorectomy, chemotherapy, radiotherapy, or hormone replacement therapy. Additionally, patients who smoked, were using vitamin supplements, or were currently pregnant or breast-feeding were excluded.

Each participant was asked to fill out a questionnaire, and the data obtained from their responses were analyzed. Trained interviewers conducted face-to-face interviews to obtain a detailed picture of the women’s social demographic data, gynecological history, lifestyle factors, and reproductive and medical history. Additionally, the age, height, weight, occupation, education, and annual household income of each participant were recorded. Anthropometric measurements (body mass and height) were performed, and BMI was calculated according to the standard formula. Normal weight was defined by a BMI range of 18.5–23.9 kg/m^2^, and obesity was defined as BMI > 30.0 kg/m^2^.

### Laboratory procedures

#### Blood collection

Blood samples were collected from all patients in the morning, after more than 8 h of fasting. Peripheral blood samples were collected from women with POI irrespective of menstrual cycle. Venous blood samples were collected from controls during the early follicular phase of the menstrual cycle (days 1–5 of a spontaneous bleeding episode) to evaluate the basal levels of FSH, luteinizing hormone (LH), estradiol (E_2_), and anti-Müllerian hormone (AMH). All samples were collected and used according to the manufacturers’ recommendations. The venous blood samples were centrifuged for 10 min at 4000 rpm at 4 °C for plasma separation. Within 30 min, the samples were transferred into airtight vials and stored at − 80 °C. Total cholesterol (TC) was measured with an enzymatic assay within 6 weeks of sample collection.

#### Hormone measurements

Serum levels of FSH, LH, E_2_, and AMH were determined using an automated Roche Modular Analytics E170 immunoassay system (Roche Diagnostics, Mannheim, Germany). Serum vitamin A levels were measured via a reverse-phase high-performance liquid chromatograph equipped with an ultraviolet detector and computerized data acquisition and storage (Pharmacia Biotech, Uppsala, Sweden). The samples were stored for < 1.5 years and were not thawed prior to analysis.

Because plasma vitamin A levels tend to change with the level of total lipids, lipid standardization is necessary to eliminate any confounding effects. Accordingly, Jordan et al. [16] and Horwitt et al. [17] suggested standardization of vitamin A levels according to the total lipid levels. Thurnham et al. [18] found that the sum of cholesterol measurements, or TC, is equivalent to the adjustment for total lipids. Therefore, to ensure that TC levels did not affect the results, the vitamin A/TC ratio was calculated.

#### Statistical analysis

Data processing and statistical analysis were performed using the SPSS software (version 17; SPSS Inc., Chicago, IL, USA). Continuous variables were presented as mean and standard deviation (SD). The Kolmogorov–Smirnov test was applied to test the normality of the data distributions (all data were presented as mean ± SD). Continuous variables with normal distributions were compared using Student’s *t*-test. Continuous variables with non-normal distribution were compared using the Mann–Whitney *U*-test. Categorical variables were presented as numbers and percentages and compared using the chi-square test. The correlations of each variable with POI were assessed with Spearman nonparametric correlation analysis. The association between the TC-adjusted vitamin A levels and POI risk was further assessed using binary logistic regression analysis. All statistical tests were two-tailed, and *p* < 0.05 was considered to indicate statistical significance.

## Results

The demographic characteristics and biochemical measurements of all the participants in this study are presented in Table 1. There were no significant differences in age and BMI at enrollment between the POI and control groups. However, women in the control group had a significantly higher annual household income and education than those in the POI group (*p* < 0.01 for both). Serum FSH and LH levels in the POI group were significantly higher than those in the control group (FSH: 72.32 ± 32.84 IU/L vs. 6.82 ± 2.26 IU/L, *p* < 0.01; LH: 39.48 ± 16.25 IU/L vs. 4.10 ± 1.51 IU/L, *p* < 0.01). Conversely, serum AMH levels were lower in the POI group than in the control group (0.02 ± 0.02 ng/mL vs. 3.36 ± 2.37 ng/mL, *p* < 0.01). Serum vitamin A levels appeared to be slightly higher in the POI group than in the control group, but there was no evidence of a statistically significant difference (728.00 ± 176.00 µg/L vs. 503.93 ± 145.64 µg/L, *p* = 0.13). However, after adjustment for serum lipid levels, the serum vitamin A/TC ratio was significantly lower in the POI group than in the control group (143.14 ± 35.86 µg/mmol vs. 157.56 ± 35.21 µg/mmol, *p* = 0.04).

**Table 1:** Clinical characteristics and biochemical parameters of the participants (*N* = 114)

Variables	POI group (*n* = 47)	Control group (*n* = 67)	*p*-Value
**Age at enrollment, n (%)**			
18–25 years	6 (12.76)	7 (10.45)	
25–29 years	7 (14.89)	15 (22.38)	
30–34 years	12 (25.53)	18 (26.86)	
35–39 years	14 (29.78)	17 (25.37)	
> 40 years	8 (17.02)	10 (14.93)	
Mean ± SD	33.30 ± 6.45	32.63 ± 5.87	0.47^a^
**BMI, n (%)**			
< 18.5 kg/m^2^	6 (12.76)	13 (19.40)	
18.5–23.9 kg/m^2^	36 (76.60)	45 (67.16)	
24.0–30 kg/m^2^	5 (10.64)	9 (13.43)	
Mean ± SD	21.11 ± 2.25	20.88 ± 2.64	0.63^b^
**Annual household income, n (%)**			
< 30,000 **RMB yuan**	7 (14.89)	1 (1.49)	< 0.01^c^
30,000–100,000 **RMB yuan**	21 (44.68)	10(14.93)	
> 100,000 **RMB yuan**	19 (40.42)	56 (83.58)	
**Education, n (%)**			
Elementary school	13 (27.65)	4 (5.97)	< 0.01^c^
High school	15 (31.91)	6 (8.96)	
College	19 (40.43)	57 (85.07)	
**Biochemical parameters**			
Mean ± SD (Range)			
FSH (IU/L)	72.32 ± 32.84 (27.86–149.47)	6.82 ± 2.26 (2.25–15.56)	< 0.01^a^
LH (IU/L)	39.48 ± 16.25 (14.33–77.56)	4.10 ± 1.51 (1.13–8.08)	< 0.01^b^
E2 (pmol/L)	188.61 ± 151.02 (43.31–755.46)	150.20 ± 74.37 (69.11–560.06)	0.37^a^
AMH (ng/mL)	0.02 ± 0.02 (0.01–0.08)	3.36 ± 2.37 (0.18–11.28)	< 0.01^a^
Vitamin A (µg/L)	728 ± 176 (349–1162)	662 ± 113 (374–943)	0.13^b^
TC (mmol/L)	5.20 ± 1.07 (3.86–9.77)	4.31 ± 0.74 (2.76–6.87)	< 0.01^b^
Vitamin A/TC	143.14 ± 35.86 (62.95–218.87)	157.56 ± 35.21 (84.04–256.16)	0.04^b^

Data are expressed as n (%) or mean ± standard deviation; *p* < 0.05 indicates statistical significance

*POI* premature ovarian insufficiency, *BMI* body mass index, *SD* standard deviation, *FSH* follicle-stimulating hormone, *LH* luteinizing hormone, *E2* estradiol, *AMH* anti-Müllerian hormone

^a^Mann-Whitney *U*-test

^b^Student’s *t*-test for independent samples

^c^Chi-square test

The POI group was divided into two subgroups based on serum FSH levels: one group with FSH level < 40 IU/L and one group with FSH level ≥ 40 IU/L (Table 2). The serum vitamin A/TC ratio was significant lower in the two POI groups than in the control group. Further, the serum vitamin A/TC ratio in the POI group with low FSH levels was lower than that in the POI group with high FSH levels, although the difference was not statistically significant (144.95 ± 39.40 µg/mmol vs. 142.77 ± 35.64 µg/mmol, *p* > 0.05).

**Table 2:** Corrected serum vitamin A levels in women with different FSH levels

	Control group	POI group	
FSH (IU/L)	< 25 (*n* = 47)	25–40 (*n* = 8)	> 40 (*n* = 39)
	Mean ± SD	Mean ± SD	Mean ± SD
Vitamin A/TC ratio	157.56 ± 35.21	144.95 ± 39.40	142.77 ± 35.64

As shown in Table 3, annual household income, education, serum AMH, and serum vitamin A/TC ratio were significantly and inversely associated with POI (*p* < 0.05 for all) according to Spearman correlation analysis. Further, the serum TC level was significantly and positively associated with POI in the study population (*p* < 0.05).

**Table 3:** Correlation of POI with confounding variables^a^

**Confounders**	**POI**	**Age**	**BMI**	**Annual household income**	**Education**	**FSH** **(IU/L)**	**LH** **(IU/L)**	**E**_**2**_ **(pmol/L)**	**AMH** **(ng/mL)**	**Vitamin A** **(µg/L)**	**TC (mmol/L)**	**Vitamin A/TC**
POI												
Age	0.06											
BMI	0.10	0.08										
Annual household income	-0.46^**^	0.10	-0.13									
Education	-0.46^**^	-0.15	-0.11	0.50^*^								
FSH (IU/L)	0.85^**^	0.22^*^	-0.04	-0.32^**^	-0.39^**^							
LH (IU/L)	0.85^**^	0.11	-0.04	-0.39^**^	-0.39^**^	0.82^**^						
E_2_ (pmol/L)	0.09	0.08	-0.08	-0.12	-0.01	0.04	0.00					
AMH (ng/mL)	-0.86^**^	-0.26^**^	-0.12	0.33^**^	0.40^**^	-0.84^**^	-0.71^*^	-0.14				
Vitamin A (µg/L)	0.21^*^	0.09	0.22^*^	-0.14	-0.19^*^	0.62^**^	0.14	0.02	-0.21^*^			
TC (mmol/L)	0.46^**^	0.20^*^	0.09	-0.19^*^	-0.26^**^	0.40^**^	0.42^*^	0.10	-0.36^**^	0.27^**^		
Vitamin A/TC	-0.20^*^	-0.07	0.14	-0.07	0.06	0.19^*^	-0.22^*^	-0.10	0.08	0.62^**^	0.54^**^	

*FSH* follicle-stimulating hormone, *LH* luteinizing hormone, *E2* estradiol, *AMH* anti-Müllerian hormone

^a^Spearman rank correlation coefficient

^*^Significant correlation at *p* < 0.05 (two-tailed)

^**^Significant correlation at *p* < 0.01 (two-tailed)

Table 4 shows the association of TC-adjusted vitamin A with POI risk determined by binary logistic regression analysis. The unadjusted odds ratio (OR) and 95% confidence interval (CI) were 0.988 and 0.977–0.999 respectively, and the association was significant (*p* = 0.04). After adjustment for age, BMI, annual household income, and education, the association remained (OR = 0.986, 95% CI: 0.972–0.999, *p* = 0.04).

**Table 4:** Association of vitamin A with POI in binary logistic regression models

Compound	Unadjusted Model		Adjusted Model ^a^	
	**OR (95% CI)**	***p*-Value**	**OR (95% CI)**	***p*-Value**
**Vitamin A/TC**	*N* = 114		*N* = 114	
	0.988 (0.977–0.999)	0.04	0.986 (0.972–0.999)	0.04

^a^The adjusted model includes age, BMI, annual household income, and education

## Discussion

In this research, we found that the serum vitamin A/TC ratio was significantly lower in women with POI than in women with normal ovarian function. Specifically, the vitamin A/TC ratio was inversely associated with POI risk. This means that vitamin A levels may be associated with the development of POI.

POI has an incidence of 1% in women and increases with age [1]. In this study, the mean age of POI patients was 33.30 ± 6.45 years, and the youngest was only 18 years old. Eighteen years is a rather young age for POI onset, and it is likely to have long-term effects on the patient. We observed that women in the control group had a significantly higher annual household income and education than those in the POI group (*p* < 0.01 for both). Similar results were found in a study in India, which reported that rural women with a low household income, nutritional deficiencies, and low educational attainment showed a higher risk of POI [19]. Based on these findings, it is important that health education be incorporated in the healthcare system to raise awareness about adequate nutrition and tackle the health problems associated with POI.

The normal reference range of serum vitamin A is 300–1200 µg/L. In the present study, the serum vitamin A levels in the POI and control groups were 728.00 ± 176.00 and 503.93 ± 145.64 µg/L, respectively. This means that no participant in our study had VAD or excessive vitamin A levels. Nonetheless, the POI patients generally showed higher serum vitamin A and cholesterol levels, while the control group showed lower serum vitamin A and cholesterol levels. Vitamin A is a fat-soluble vitamin, and its plasma levels changes with total lipid levels. Therefore, we used the vitamin A/TC ratio to eliminate the confounding effects of lipids in evaluating the role of vitamin A in POI. The serum vitamin A/TC ratio was significantly lower in the women with POI than that in controls (143.14 ± 35.86 µg/mmol vs. 157.56 ± 35.21 µg/mmol, *p* < 0.05). It is believed that a serum FSH level of ≥ 40 IU/L is a marker of late-stage POI [20]. In this study, we found that the serum vitamin A/TC ratio in the POI group with FSH levels < 40 IU/L was higher than that in the POI group with FSH levels ≥ 40 IU/L. It is proved that the increase of serum FSH level is related to the decrease of vitamin a level. Thus, our results seem to indicate that increase in the severity of POI may be associated with decreasing vitamin A levels.

Our findings also indicated that annual household income, education, serum AMH levels, and serum vitamin A/TC ratio were significantly and inversely associated with POI risk. Further, binary logistic regression analysis demonstrated a significant association between TC-adjusted vitamin A levels and POI risk (OR = 0.988, 95% CI: 0.977–0.999, *p* = 0.04). After adjusting for annual household income, serum AMH levels, and education, the association remained (OR = 0.986, 95% CI: 0.972–0.999, *p* = 0.04). These results indicate that the serum vitamin A/TC ratio could act as a predictor of POI incidence. Kazami et al. found that dietary antioxidant vitamin intake improved oocyte competence [21]. Therefore, vitamin A may play a protective role in ovarian tissue, and increasing the intake of vitamin A-rich foods or using vitamin A nutritional supplements may help in the prevention and treatment of POI.

A limitation of our study is the relatively small sample size, but the results of this study are still encouraging. Because of the relatively small number of cases, further studies with a large number of patients are necessary to validate these findings. Second, the serum vitamin A levels we detected may not reflect ovarian vitamin A levels. In the future, the results should be validated by directly measuring vitamin A levels in ovarian tissue in order to evaluate the correlation between vitamin A and ovarian function in the future. Third, although this study found that the serum vitamin A/TC ratio was inversely and significantly associated with the risk of POI, the difference in vitamin A levels between the groups was still small. Therefore, as mentioned earlier, further studies with large sample sizes are needed to validate the present findings.

## Conclusion

In conclusions, our study revealed an inverse association between TC-corrected serum vitamin A levels and POI risk, but the findings warrant further research in a larger population. Despite this, there is some implication that adequate vitamin A supplementation may help prevent or delay POI development.

## Data Availability

The datasets used and/or analyzed during the current study are available from the corresponding author on reasonable request.

## Abbreviations

AMH: Anti-Müllerian hormone
BMI: Body mass index
E2: Estradiol
FSH: Follicle-stimulating hormone
LH: Luteinizing hormone
POI: Premature ovarian insufficiency
TC: Total cholesterol
VAD: Vitamin A deficiency
